# The early impact of COVID-19 vaccination on deaths among elderly people in Iran 

**Published:** 2022

**Authors:** Seyed Amir Ahmad Safavi-Naini, Mohamad Amin Pourhoseingholi

**Affiliations:** 1 *National Research Institute of Tuberculosis and Lung Diseases, Shahid Beheshti University of Medical Sciences, Tehran, Iran*; 2 *Basic and Molecular Epidemiology of Gastrointestinal Disorders Research Center, Research Institute for Gastroenterology and Liver Diseases, Shahid Beheshti University of Medical Sciences, Tehran, Iran*

 In the coronavirus disease 2019 (COVID-19) pandemic, effective vaccination was one of the leading solutions to limit fatalities from COVID-19. Iran started COVID-19 vaccination on 10 February 2021 for frontline healthcare workers (HCW). Then patients with cancer, thalassemia, renal failure, and multiple sclerosis were vaccinated in the first phase of Iran’s vaccination program. The second phase which aimed to vaccinate the elderly population started in April 2021, and on 29 June 2021, 1,641,010 people had been fully vaccinated (2% of the total population) (1, 2). The 70+-year-old population had access to COVID-19 vaccines until the end of spring 2021, and in early summer, online registration was available for those under 70 years of age (3).

The fourth wave of COVID-19 hit Iran in the summer of 2021 and infected more than 2 million people; thence, some concerns were raised about the effectiveness of the vaccination program. The impact of vaccination on fatality remained obscure, and the lack of evidence led anti-vaxxers to question the efficacy of COVID-19 vaccination. Here, we aimed to estimate the changes in all-cause deaths in the 70+-year-old population after vaccination in spring 2021. Death statistics and data on reported COVID-19 cases were gathered from publicly available datasets (4). Autoregressive integrated moving average with explanatory variable (ARIMAX) (5) was used to estimate the predicted monthly all-cause deaths in 5-year increment age groups and its 95% confidence interval. The Solar Hijri monthly all-cause deaths from 21 March 2020 to 21 June 2021 were used to predict the monthly all-cause deaths from 22 June 2021 to 22 September 2021, assuming reported COVID-19 cases as an explanatory variable. Beforehand, a significant regression equation was found between reported COVID-19 cases and monthly all-cause mortality and was statistically significant (F (1,15) =12.97, *p*=0.03, R^2^=0.42).

In summer 2021, because of the deadly fifth wave of COVID-19 disease, all-cause mortality reached 162,570 deaths. Compared to summer 2019 with 98,722 deaths, excess deaths in summer 2021 were 63,848 and evident in all age groups. As depicted in Figure 1, observed death counts were higher than the predicted value in under 70-year-olds, even after assuming the COVID-19 case count as an explanatory variable. Nevertheless, the number of all-cause deaths were lower 

**Table 1:** The monthly observed-predicted death gap in older than 70-years-old population during summer 2021

Solar Hijri Month (Georgian date)	Tir (22 June-22 July)	Mordad (23 July-22 August)	Shahrivar (23 August-22 September	Summer (22 June-22 September)
COVID-19 reported cases	518,220	1,053,274	800,115	2,371,609
Predicted-observed death gap in older than 70 group	-8,526 (-18,467-1,414)	-1,104 (-11,044-8,836)	-2,671 (-12,612-7,269)	-12,302 (-42,122-17,519)
Predicted-observed death gap in younger than 70 group	-4,845 (-11,732-2,042)	9,285 (2,398-16,172)	5,494 (-1,393-12,381)	9,934 (-10,727-30,595)
Percent of death gap in older than 70s to predicted deaths in older than 70s	-31.4 (-67.9- 5.2)	-3.3 (-32.7- 26.2)	-8.7 (-41.1- 23.7)	-13.4 (-46- 19.1)
Percent of death gap in older than 70s group to predicted deaths in all-age groups	-17.3 (-37.4 to 2.9)	-1.8 (-18.3 to 14.6)	-4.8 (-22.9 to 13.2)	-7.5 (-25.5 to 10.6)

**Figure 1:**
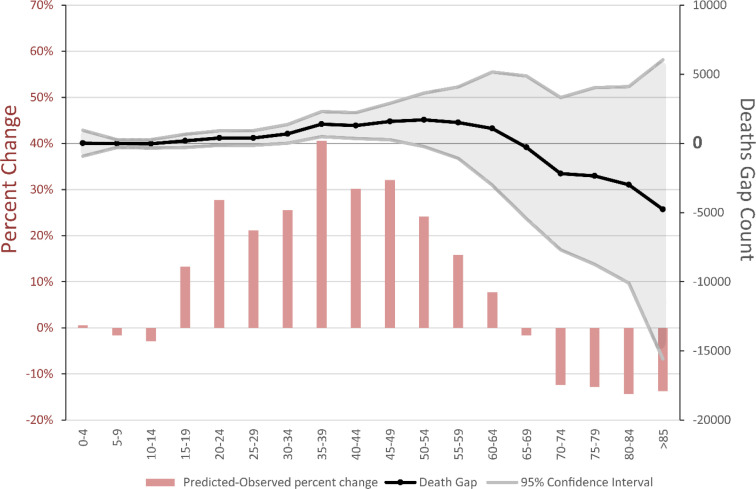
The predicted-observed gap and percent change of all-cause mortality disaggregated by age-groups in summer 2021

than expected in the over 70-year-old population, the population who received the COVID-19 vaccine in spring (3). The reduced death rate per 100,000 people over 70 years of age was 369.9 in summer 2021. The 12,302 fewer deaths in the over 70-year-old population were 19% and 7.5% of total excess deaths and total deaths of summer 2021, respectively (Table 1). 

In line with our results, the vaccination of the over 80-year-old Brazilian population prevented 13,824 deaths compared to the predicted value (6). Moreover, most of the reduction in deaths in the UK and the USA was attributed to the vaccination of the elderly population (7, 8). However, the real impact of vaccination on mortality may be beyond our estimation, as is the impact of the COVID-19 pandemic. The reduced number of deaths in the older than 70 years population can be explained by the direct effect of vaccines on mortality from COVID-19 and the indirect effects of vaccination on non-COVID-19 mortality (9). The outbreak of COVID-19 cases may lower the efficacy of COVID-19 vaccines (10). We further found that the minimum reduction of mortality occurred in the month with the maximum infection of COVID-19 (Table 1). 

Here we approximated the impact of vaccination of the elderly population on all-cause deaths based on the limited available data. More studies are needed to investigate the vaccination impact on COVID-19 and other causes of death. Still, vaccination of the elderly had a significant impact on all-cause mortality in Iran.

## Conflict of interests

The authors declare that they have no conflict of interest.
